# The Evidence-Based Approach to Adult-Onset Idiopathic Nephrotic Syndrome

**DOI:** 10.3389/fped.2015.00078

**Published:** 2015-09-25

**Authors:** Pietro A. A. Canetta, Jai Radhakrishnan

**Affiliations:** ^1^Division of Nephrology, Department of Medicine, Columbia University Medical Center, Columbia University College of Physicians and Surgeons, New York, NY, USA

**Keywords:** nephrotic syndrome, minimal-change disease, focal segmental glomerulosclerosis, KDIGO guidelines, clinical practice guidelines

## Abstract

Adult-onset nephrotic syndrome (NS) differs from its pediatric counterpart in several important ways. Most importantly, NS in adults is more etiologically heterogeneous compared to children, and thus treatment approaches rely heavily on the histological diagnosis provided by renal biopsy. The evidence-based approach to treatment of adult NS has been critically examined by the Kidney Disease Improving Global Outcomes (KDIGO) guidelines in glomerulonephritis, published in 2012. Here, we examine the strengths and limits of those guidelines and review recent work that expands the evidence-based approach.

## Introduction

The field of nephrology carries the dubious distinction of consistently producing the fewest number of randomized controlled trials among the internal medicine specialties (1, 2). Within nephrology, glomerular diseases have been particularly neglected, especially the primary glomerular diseases, including idiopathic nephrotic syndrome (NS) (3, 4). Consequently, high-quality clinical evidence to guide treatment decisions in NS has been sparse, and nephrologists have long navigated these murky waters according to principles of physiology and insights gleaned from observational or uncontrolled studies.

With these limitations in mind, a Kidney Disease Improving Global Outcomes (KDIGO) working group attempted to synthesize the available evidence into a series of disease-specific clinical practice guidelines for glomerulonephritis, published in 2012 (5). These guidelines were pioneering in that nothing so comprehensive with respect to glomerular diseases had ever been produced by one of the field’s major organizations. Other systematic reviews have examined specific diseases or problems in NS [particularly noteworthy are Cochrane reviews covering corticosteroid use, immunosuppression, and lipid-lowering therapy (6–8)], but none claimed broad consensus to establish practice guidelines. Clinical practice guidelines serve several important purposes to clinicians and researchers (9). By critically synthesizing and grading the quality of the evidence base, they help define not only what is optimal but also the breadth of what is reasonable. By defining the limits of current evidence, they highlight those areas where evidence is lacking to justify adoption or broad endorsement of specific practices.

Using the KDIGO guidelines as a foundation, we here examine the evidence-based approach to adult-onset NS, limiting the discussion to minimal-change disease (MCD) and focal segmental glomerulosclerosis (FSGS). Strikingly, of the recommendations offered in the KDIGO guidelines, over 75% were considered to be based on low- or very low-quality evidence (grade C and D, respectively). In particular, for adult MCD and adult FSGS, a total of 23 statements were made of which only 2 (8.7%) were level 1 recommendations, both based on grade C evidence. No statement was supported by grade A evidence, and only one suggestion was graded B (moderate quality). Of the remaining 20 statements, 17 were classified as suggestions based on low- or very low-quality evidence (2C or 2D), and three statements (13%) lacked evidence to be graded at all. This is a sobering picture of the limits of evidence that define the “state of the art” in the clinical management of adult NS.

## Comparing Adult-Onset to Pediatric Nephrotic Syndrome

Adult-onset NS differs from its pediatric counterpart in several important ways. The prospective International Study of Kidney Diseases in Children showed that in children aged 1–16 presenting with NS, there was a 95% likelihood of responding to a course of glucocorticoids by 16 weeks (10). This finding justified the recommendation that kidney biopsy not be routinely done immediately in nephrotic children, but rather deferred to those not responding to (or relapsing after) an empiric course of corticosteroids. Importantly, most children presenting with NS will have MCD, although the incidence of other diagnoses appears to be increasing (particularly FSGS) and these account for most treatment-resistant cases (11, 12). In adults, NS comprises a much more diverse group of diseases, with considerable demographic variation, including both primary and secondary conditions (13, 14). For this reason, early kidney biopsy is critical to properly categorize the disease and direct the subsequent clinical approach in adults. Among primary or idiopathic diseases, the most common remain FSGS, membranous nephropathy, and MCD. Because this Frontiers research topic is primarily concerned with pediatric NS, we will focus on MCD and FSGS, which also cause the majority of pediatric NS and where comparisons may be most fruitful.

The prevalence of genetic causes of NS is unsurprisingly different between pediatric and adult populations. An increasing number of monogenetic defects have been found to cause neonatal or childhood NS, most of which affect genes critical to podocyte function, follow autosomal recessive transmission, and histologically produce FSGS (15, 16). The precise role for genetic screening remains ill-defined. The KDIGO guidelines did not endorse screening, stating that

Routine evaluation for genetic mutations is not recommended in this guideline due to the variable availability of genetic testing, significant cost, low to absent prevalence observed in some populations, and the lack of systematic studies of treatment response and prognosis relative to specific genetic polymorphisms (5)

Reconsideration of such claims should be prompted by the pace of discovery and technological improvements in genetic diagnosis, precipitously falling costs, expanding availability, and progress in understanding the clinical consequences of mutations.

Genetic variants may predict with high accuracy which patients will have steroid-resistant disease, potentially allowing a clinician to spare them from toxic empiric therapy (17). Emerging research suggests that there may also be a genetic signal for steroid-*sensitive* disease (18). Certain mutations also predict freedom from relapse following transplantation (15). Mutations are increasingly being found in older patients with NS. A recent publication from the SRNS Study Group showed that a single-gene cause of steroid-resistant NS could be identified in nearly a third of affected families, including >10% where disease onset was between 13 and 18 years of age (19). Santín and colleagues likewise identified a high proportion of mutations in Spanish patients with primary NS, including 14% among adults (median age of onset 33 years), and proposed a screening algorithm based on age of onset and whether disease was familial or sporadic (20). Such algorithms will need to be validated and adjusted based on local demographics, testing availability, and reimbursement practices, but with the advancements in speed and affordability of next-generation sequencing there is already reasonable justification for screening selected patients in the clinic.

## Adult Minimal-Change Disease

Adult MCD shares many similarities to the pediatric form, although time to remission appears prolonged and acute kidney injury more common, whereas the risk of relapse may be less (21–23). The largest published series of adult MCD included 340 Chinese patients (24). Only 9.7% were steroid resistant, the remainder reached remission in a median of 10 weeks. During follow-up, 42% of responders relapsed, and 27% became frequent relapsers or steroid dependent (FR/SD). Certain groups may have higher morbidity. In a series of 95 adults seen at our referral center, almost all treated with steroids, 25% presented with acute kidney injury (25). Mean time to remission was 13 weeks, but one-quarter were steroid-resistant and 73% of responders relapsed, with 41% of responders frequently relapsing.

The KDIGO guidelines for adult MCD are presented in Box 1. Several recommendations deserve discussion. Like in pediatric NS, corticosteroids are recommended as first-line therapy (graded 1C). Clinicians will recognize that the dose of corticosteroids is high enough to cause significant morbidity, such as Cushings syndrome, bone loss, and hyperglycemia, especially over the time suggested (minimum 4 weeks, maximum 16 weeks, with a slow taper up to 6 months). Is it possible to achieve results with less? Direct evidence from adults is largely lacking, but at least three recent randomized controlled studies in children showed that extended courses of steroids did not prevent the development of FR/SD disease, just delayed its recognition (26–28). This prompted a revision to a Cochrane systemic review, concluding that the benefit of prolonged courses of steroids was likely overestimated by earlier studies and that it seems there is no benefit of increasing the duration of prednisone beyond 2–3 months (6). Should these pediatric studies influence practice in adults? Here, it is worth noting that the KDIGO guidelines are based on low-quality evidence, and indeed their “recommendation is based *largely on extrapolation from RCTs in children*” (p. 177, emphasis ours).

Box 1 **KDIGO guidelines for minimal-change disease (MCD) in adults.**

5.1: *Treatment of initial episode of adult MCD*

5.1.1: We recommend that corticosteroids be given for initial treatment of nephrotic syndrome. (*1C)*

5.1.2: We suggest prednisone or prednisolone be given at a daily single dose of 1 mg/kg (maximum 80 mg) or alternate-day single dose of 2 mg/kg (maximum 120 mg). (2*C*)

5.1.3: We suggest the initial high dose of corticosteroids, if tolerated, be maintained for a minimum period of 4 weeks if complete remission is achieved, and for a maximum period of 16 weeks if complete remission is not achieved. (*2C*)

5.1.4: In patients who remit, we suggest that corticosteroids be tapered slowly over a total period of up to 6 months after achieving remission. (*2D*)

5.1.5: For patients with relative contraindications or intolerance to high-dose corticosteroids (e.g., uncontrolled diabetes, psychiatric conditions, severe osteoporosis), we suggest oral cyclophosphamide or CNIs as discussed in frequently relapsing MCD. (*2D*)

5.1.6: We suggest using the same initial dose and duration of corticosteroids for infrequent relapses as in Recommendations 5.1.2, 5.1.3, and 5.1.4. (*2D*)

5.2: *FR/SD MCD*

5.2.1: We suggest oral cyclophosphamide 2–2.5 mg/kg/day for 8 weeks. (*2C*)

5.2.2: We suggest CNI (cyclosporine 3–5 mg/kg/day or tacrolimus 0.05–0.1 mg/kg/day in divided doses) for 1–2 years for FR/SD MCD patients who have relapsed despite cyclophosphamide, or for people who wish to preserve their fertility. (*2C*)

5.2.3: We suggest MMF 500–1000 mg twice daily for 1–2 years for patients who are intolerant of corticosteroids, cyclophosphamide, and CNIs. (*2D*)

5.3: *Corticosteroid-resistant MCD*

5.3.1: Re-evalulate patients who are corticosteroid resistant for other causes of nephrotic syndrome. (*Not Graded*)

5.4: *Supportive therapy*

5.4.1: We suggest that MCD patients who have AKI be treated with renal replacement therapy as indicated, but together with corticosteroids, as for a first episode of MCD. (*2D*)

5.4.2: We suggest that, for the initial episode of nephrotic syndrome associated with MCD, statins not be used to treat hyperlipidemia, and ACE-I or ARBs not be used in normotensive patients to lower proteinuria. (*2D*)

FR, frequently relapsing; SD, steroid dependent; CNI, calcineurin inhibitor; MMF, mycophenolate mofetil; AKI, acute kidney injury.

A separate issue concerns the recommendations for alternative agents to corticosteroids. KDIGO suggests alternative agents in patients with relative contraindications or intolerance to high-dose corticosteroids (2D), or for patients with FR/SD disease (2C/2D). Three agents are named: cyclophosphamide and calcineurin inhibitors (CNIs) receive slightly stronger endorsement, followed by mycophenolate mofetil (MMF). Several points here deserve mention. One consideration is why these agents are considered second-line behind steroids. There have been only two controlled trials of steroids in adult MCD, only one placebo-controlled (the other comparator was no treatment), involving, respectively, 28 and 31 subjects (29). Steroids have never been directly compared against alternate agents for initial treatment, and observational data suggests similar (if not better) frequencies of response to alternate agents (21, 22, 25). The strength of recommendation supporting steroids rests largely on the totality of evidence showing that steroids are effective (especially in children), but NOT on evidence showing that other agents are ineffective or that they are inferior to steroids. The reason steroids have been studied more is that they have been used more, largely for historical and circumstantial reasons (availability, affordability, prescriber familiarity, etc.). There is certainly sufficient equipoise to justify a randomized trial in adults comparing an alternative (and one hopes better tolerated) agent to corticosteroids, though only time will tell if the nephrology community deems this a sufficient priority to pursue.

Recommendation 5.1.5 to use alternate agents for patients with contraindications or intolerance to steroids is graded as 2D, a suggestion based on low-quality evidence. It should be recognized that the weakness of the evidence relates to the choice of replacement, not to the premise of withholding steroids in the first place – there need be very little “evidence” to justify NOT giving a medication which is poorly tolerated or contraindicated! In adults, relative contraindications to steroid use are more common (e.g., diabetes, osteoporosis), as is steroid intolerance, and in clinical decision-making individual patient considerations must trump broad practice guidelines (30). Another nuance not specifically considered in the guidelines, but reflected by the weak grading of evidence, concerns the long-term sequelae of various therapeutic choices. The justification for rating cyclophosphamide and CNIs as nearly equivalent in the guidelines is based on observational studies and a few small randomized trials comparing the two [summarized in the guidelines and elsewhere (29)]. These studies have no information on potential long-term toxicities of the drugs, for example, late malignancies from cyclophosphamide or nephrotoxicity from CNIs. Obtaining such evidence is certainly feasible; for instance, it was recently shown in a carefully followed cohort of membranous nephropathy (another common cause of idiopathic NS in adults) that treatment with cyclophosphamide was associated with a more than threefold increased risk of subsequently developing cancer (31).

The anti-CD20 monoclonal antibody rituximab is not mentioned in the KDIGO guidelines on adult MCD, except in an appeal for further research. In the years since the guidelines were published, such research increasingly supports the efficacy of rituximab in both children and adults, particularly for steroid sensitive and SD/FR disease. The Rituximab for Childhood-onset Refractory Nephrotic Syndrome Study in Japan was a multicenter, double-blind, randomized trial of rituximab vs. placebo in 52 children with FR/SD NS (32). Median relapse-free survival was 267 days in the rituximab group compared to 101 days in the placebo group (*P* < 0.0001). The rituximab group had significantly fewer relapses, and required significantly less steroids. In Italy, Ravani and colleagues carried out a multicenter, open-label, randomized non-inferiority trial of rituximab vs. tapering steroid therapy in 30 children with SD NS on high-dose prednisone (33). The differences in relapse were dramatic; median time to relapse was 18 months for the rituximab group, whereas 14/15 children in the control group relapsed within 4 months. The rituximab group received much lower cumulative doses of prednisone. Observational studies bolster the results of these randomized studies. Recent reports from large case series in children, and several series of adults with MCD, have consistently found that rituximab is associated with prolonged remissions and allows reduction or cessation of steroids or other immunosuppressants in SD/FR patients (34–38). In sum, these data support a role for rituximab in the care of patients with SD/FR disease. Where rituximab should rank among the various other immunosuppression choices may be clarified by future head-to-head clinical trials and careful cost-benefit analyses.

## Adult Idiopathic FSGS

The KDIGO guidelines for adult FSGS are presented in Box 2. Many of the treatment recommendations regarding steroid use are analogous to those for MCD and recapitulate the issues discussed earlier. Some additional issues specific to FSGS are worth exploring further.

Box 2 **KDIGO guidelines for idiopathic focal segmental glomerulosclerosis (FSGS) in adults.**

6.1: *Initial evaluation of FSGS*

6.1.1: Undertake thorough evaluation to exclude secondary forms of FSGS. (*Not Graded*)

6.1.2: Do not routinely perform genetic testing. (*Not Graded*)

6.2: *Initial treatment of FSGS*

6.2.1: We recommend that corticosteroid and immunosuppressive therapy be considered only in idiopathic FSGS associated with clinical features of the nephrotic syndrome. (*1C*)

6.2.2: We suggest prednisone be given at a daily single dose of 1 mg/kg (maximum 80 mg) or alternate-day dose of 2 mg/kg (maximum 120 mg). (*2C*)

6.2.3: We suggest the initial high dose of corticosteroids be given for a minimum of 4 weeks; continue high-dose corticosteroids up to a maximum of 16 weeks, as tolerated, or until complete remission has been achieved, whichever is earlier. (*2D*)

6.2.4: We suggest corticosteroids be tapered slowly over a period of 6 months after achieving complete remission. (*2D*)

6.2.5: We suggest CNIs be considered as first-line therapy for patients with relative contraindications or intolerance to high-dose corticosteroids (e.g., uncontrolled diabetes, psychiatric conditions, severe osteoporosis). (*2D*)

6.3: *Treatment for relapse*

6.3.1: We suggest that a relapse of nephrotic syndrome is treated as per the recommendations for relapsing MCD in adults (see Chapters 5.1 and 5.2). (*2D*)

6.4: *Treatment for steroid-resistant FSGS*

6.4.1: For steroid-resistant FSGS, we suggest that cyclosporine at 3–5 mg/kg/day in divided doses be given for at least 4–6 months. (*2B*)

6.4.2: If there is a partial or complete remission, we suggest continuing cyclosporine treatment for at least 12 months, followed by a slow taper. (*2D*)

6.4.3: We suggest that patients with steroid-resistant FSGS, who do not tolerate cyclosporine, be treated with a combination of mycophenolate mofetil and high-dose dexamethasone. (*2C*)

CNI, calcineurin inhibitors.

The first two recommendations, 6.1.1 and 6.1.2, are not graded and present a small paradox. The clinician is urged to perform a thorough evaluation to exclude secondary forms of FSGS, but what this evaluation should entail is left undefined except that it should *not* include genetic testing. Following these non-graded recommendations is a relatively strongly graded (1C) recommendation that corticosteroids or immunosuppression be considered *only* for “idiopathic FSGS with clinical features of nephrotic syndrome.” It is not specified which particular clinical features of NS are sufficient to justify therapy.

The guidelines are necessarily vague because of the inherent limitations in our classification of FSGS. It should be clear that the purpose of these recommendations is to direct steroids and immunosuppression to those patients with the highest likelihood of response (idiopathic FSGS), while excluding those for whom such therapy is ineffective (most cases of secondary FSGS). However, establishing a clear diagnosis of idiopathic FSGS – and by extension, deciding whom to treat with steroids/immunosuppression – is not trivial. There is no evidence-based approach for ruling out secondary FSGS. The list of conditions that may produce FSGS lesions on biopsy is long and diverse (39). In the rationale, the KDIGO guidelines state, “idiopathic FSGS is defined by exclusion of *any other identifiable cause* of secondary FSGS” (p. 181, emphasis ours), but this definition is problematic. Obesity, diabetes, and hypertension all may cause secondary FSGS, but these are common conditions and none of them exclude the possibility of a separate or superimposed “idiopathic” FSGS. Such issues present real, practical challenges to the clinician attempting to apply the guidelines in deciding whether to treat a patient with nephrotic-range proteinuria and a biopsy showing FSGS lesions.

These concerns highlight the limitations of classifying FSGS as “idiopathic” or “secondary.” At the heart of the matter is determining whether a patient’s cause of FSGS may be susceptible to steroids or immunosuppression, given the overwhelming evidence that achieving partial or complete remission with therapy dramatically improves prognosis (40–44). Identifying the circulating factor or factors responsible for idiopathic FSGS remains a yet unrealized hope, but one that when achieved should greatly assist in determining which patients deserve a trial of immunotherapy (as well as chipping away at the inelegant term, “idiopathic.”) In the meantime, what evidence is available to help determine whom to treat?

A potentially evolving role for genetic testing was discussed previously, and in a future of personalized medicine may become a routine part of the diagnostic workup. Some additional points may be helpful. Deegens and colleagues showed that the degree of foot process effacement, measured by electron microscopy, was a sensitive and specific test to differentiate idiopathic from secondary FSGS (45). In fact, foot process effacement seen in post-reperfusion biopsies of transplanted kidneys diagnosed recurrent FSGS with high specificity (46). By light microscopy, the perihilar variant of FSGS is most characteristic of hyperfiltration injury, but does not rule out idiopathic FSGS (47). Among clinical features, normal serum albumin and lack of edema despite nephrotic-range proteinuria are common findings in secondary FSGS, but rare in idiopathic FSGS (48). In the absence of nephrotic-range proteinuria, immunosuppression is generally unnecessary since subnephrotic patients have excellent long-term renal prognosis with kidney survival rates exceeding 90% at 5–10 years on conservative therapy alone (43, 49, 50).

The treatment of steroid-resistant FSGS remains particularly challenging. KDIGO suggests cyclosporine with a 2B recommendation. Tacrolimus, the other commonly used CNI, is not formally recommended. The rationale cites the lack of randomized trials, but also states that limited observational data suggests tacrolimus may be an alternative to cyclosporine. Additional data published after the KDIGO guidelines support this notion. A small, single-center study of adults with idiopathic FSGS compared intravenous monthly cyclophosphamide to tacrolimus for 6 months, with both groups receiving steroids (51). There were no significant differences between the groups, but both groups had improved proteinuria and serum albumin with stable GFR. Tacrolimus was also compared to cyclophosphamide in a multicenter randomized trial of 131 children with steroid-resistant NS (mostly MCD and FSGS) (52). Tacrolimus showed remarkably higher rates of complete or partial remission (82.5 vs. 45.9%, *P* < 0.001), with shorter time to remission and fewer serious infections. The superior likelihood of remission was maintained in the subgroup with FSGS (HR 2.54, 95% CI 1.09–5.93, *P* = 0.03). In an uncontrolled trial of 44 adults with steroid-resistant FSGS treated with tacrolimus for 24 weeks, 52.3% of patients achieved complete or partial remission (53).

For steroid-resistant patients intolerant to cyclosporine, KDIGO recommends only combined MMF and high-dose dexamethasone. This is based on one large randomized trial which reported that this combination had similar efficacy to cyclosporine, but the limitations of this trial have been reviewed in detail (54). No other agents receive formal recommendations; in our view, this is largely appropriate for practice guidelines given the limited evidence concerning other agents. Of note, no formal recommendation is made for or against cytotoxics, although they are explicitly discouraged in the KDIGO guidelines for steroid-resistant NS in children (5). Rituximab has shown potential for steroid-sensitive FSGS in case reports and small series, but it appears largely ineffective for steroid-resistant disease (55–57). A recent small series examined adrenocorticotropic hormone gel in 24 patients with idiopathic FSGS and found that 7 (29%) experienced remission with therapy, including 5/15 (33%) who were steroid-resistant.

**Figure 1:**
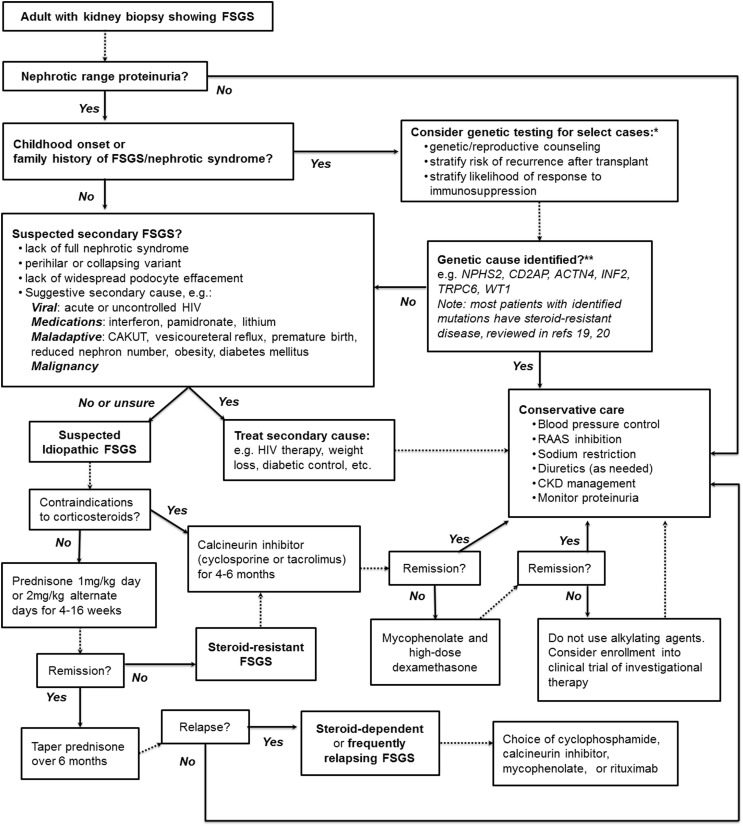
**A suggested algorithm for the clinical approach to adult FSGS**. *Note that KDIGO guidelines explicitly recommend against genetic testing. **Highlighted are some of the monogenic causes of FSGS more likely to be found in adults with genetic disease; see text and highlighted references for further discussion. *NPHS2*, Podocin; *CD2AP*, CD2-associated protein; *ACTN4*, α-actinin-4; *INF2*, inverted formin-2; *TRPC6*, transient receptor potential channel 6; *WT1*, Wilms tumor protein.

Figure 1 presents our suggested summary algorithm for the diagnostic and therapeutic approach to an adult with FSGS on kidney biopsy, which largely follows the KDIGO guidelines. Such an algorithm, like the practice guidelines themselves, should not supplant clinical judgment.

## Conclusion

The KDIGO clinical practice guidelines have played a pioneering role in providing evidence-based consensus recommendations for treating glomerular diseases, including adult idiopathic NS. While the recommendations are necessarily limited by the quality of evidence underlying them, they serve an excellent starting point for a discussion of the evidence-based approach to NS. New research adds to, but rarely supplants, prior evidence, which is why venerable “tried and true” interventions such as corticosteroids continue to play such a prominent role in the recommendations. As with all clinical practice guidelines, a critical appreciation of their limitations and an eye toward emerging lines of evidence are necessary to most effectively apply their lessons to individual patients.

## Author Contributions

PC drafted the manuscript. JR conceptualized the work and critically revised it.

## Conflict of Interest Statement

Pietro A. A. Canetta declares no commercial or financial relationships that could be construed as potential conflicts of interest. Jai Radhakrishnan was a member of the work group that formulated the KDIGO Clinical Practice Guideline for Glomerulonephritis.
